# A case study of interventions to facilitate learning for pupils with hearing impairment in Tanzania

**DOI:** 10.4102/ajod.v11i0.974

**Published:** 2022-11-10

**Authors:** Tron V. Tronstad, Bjørn Gjessing, Ingvild Ørland, Tone Øderud, Cosmas Mnyanyi, Isaack Myovela, Jon Øygarden

**Affiliations:** 1 Digital Department, SINTEF, Trondheim, Norway; 2 Department of Neuromedicine and Movement Science, Faculty of Medicine and Health Sciences, Norwegian University of Science and Technology, Trondheim, Norway; 3 Department of Otorhinolaryngology, Lovisenberg Diaconal Hospital, Oslo, Norway; 4 Department of Psychology and Special Education, Faculty of Education, Open University of Tanzania, Dar Es Salaam, Tanzania; 5 Department of Hearing Impairment, Patandi College of Special Needs and Inclusive Setting, Arusha, Tanzania

**Keywords:** hearing impairment, personal sound amplification system, speech-in-noise test, hearing interventions, school children

## Abstract

**Background:**

Hearing is essential for learning in school, and untreated hearing loss may hinder quality education and equal opportunities. Detection of children with hearing loss is the first step in improving the learning situation, but effective interventions must also be provided. Hearing aids can provide great benefit for children with hearing impairment, but this may not be a realistic alternative in many low- and middle-income countries because of the shortage of hearing aids and hearing care service providers.

**Objective:**

In this study, alternative solutions were tested to investigate the potential to improve the learning situation for children with hearing impairment.

**Method:**

Two technical solutions (a personal amplifier with and without remote microphone) were tested, in addition to an approach where the children with hearing impairment were moved closer to the teacher. A Swahili speech-in-noise test was developed and used to assess the effect of the interventions.

**Results:**

The personal sound amplifier with wireless transmission of sound from the teacher to the child gave the best results in the speech-in-noise test. The amplifier with directive microphone had limited effect and was outperformed by the intervention where the child was moved closer to the teacher.

**Conclusion:**

This study, although small in sample size, showed that personal amplification with directive microphones did little to assist children with hearing impairment. It also indicated that simple actions can be used to improve the learning situation for children with hearing impairment but that the context (e.g. room acoustical parameters) must be taken into account when implementing interventions.

**Contribution:**

The study gives insight into how to improve the learning situation for school children with hearing impairment and raises concerns about some of the known technical solutions currently being used.

## Introduction

Globally, there are about 466 million people (6.1% of the world’s population) with hearing loss (HL), of which approximately 34 million are children (World Health Organization 2020). Nearly 90% of people with HL live in low- and middle-income countries (LMICs), often lacking the resources and services to address HL (World Health Organization 2018).

Hearing loss may be mild, moderate, severe or profound and can affect one or both ears. Without a systematic approach of detecting HL, only those with more severe HL are detected, often by the community (guardians, teachers, health workers and peers). This means that persons with mild to moderate HL often go undetected, even if such HL still leads to difficulty in hearing conversational speech (World Health Organization 2020). As listening is a main form of learning, children with HL often have lower school performance than children without HL (Flexer, Millin & Brown 1990; Lieu et al. 2010). In many LMICs, children with HL and deafness are vulnerable to dropping out of school, not achieving expected learning goals or never going to school, with girls being more at risk of dropping out or never attending (Njelesani et al. 2018; UNICEF n.d.; World Health Organization 2020). Lack of education affects adult life with respect to obtaining and maintaining employment.

Public awareness about childhood HL in LMICs is often poor and often aggravated by negative attitudes, superstition, traditional customs and cultural beliefs (Swanepoel, Störbeck & Friedland 2009). Children with disabilities, including HL, are therefore more vulnerable to physical, social, emotional and sexual abuse and even murder (Njelesani et al. 2018; Olusanya, Neumann & Saunders 2014). As undetected HL is an ‘invisible’ impairment, children are often misunderstood as slow learners or impudent when they do not respond to questions or requests. This was exemplified by Dr Olusanya in an interview given in 2019. She was born in Nigeria with a mid-frequency HL that was not detected until she was an adult. She remembered growing up angry because of frequent and unjustified punishment for not doing as she was told, even though she always did everything that she could hear (Cousins 2019).

In an ongoing project in Tanzania, the prevalence of HL among school children was assessed. In 2019, the prevalence was found to be between 7% and 17% of school children in Kilimanjaro, Tanzania (Solvang et al. 2020). A review of prevalence studies from 1993 to 2012 in a range of African countries reported similar numbers (3% – 21%), indicating that the situation had not changed for decades (Mulwafu, Kuper & Ensink 2016). The prevalence of HL in children in LMICs is substantial, and establishing hearing care services for these children can help millions to achieve a better education.

An estimated 75% of HL in children under 15 living in LMICs is preventable (World Health Organization 2020). This was supported by the Kilimanjaro study mentioned here, where 58% of the children with HL had impacted earwax or foreign bodies in their ears and 31% had ear infections (Solvang et al. 2020). The literature review by Mulwafu et al. (2016) also reported that the most common cause of HL was middle ear disease (36%), followed by undetermined causes (35%) and earwax blocking the ear canal (24%). Unfortunately, in most LMICs, including Tanzania, children are not screened for HL and preventive measures are rarely accessible.

In the ongoing project reported in this article, the goal is to develop a sustainable hearing screening programme for school children in Tanzania. Identifying children with HL is the first step towards improving their learning situation. However, it is important to observe that detection alone is not sufficient to solve the problem. A study in Malawi found that only 3% of the children found with HL attended their referral appointment with an ear and hearing service (Bright et al. 2017). The most common causes for not attending were found to be transport difficulties, lack of information regarding the referral and financial constraints. The indirect cost associated with, for example, transport and food has been found to be a substantial barrier to persons attending healthcare sessions, even in countries with free medical care (Bright et al. 2017; Mahande et al. 2007). A follow-up of the Malawi study found that counselling by a trained community health worker and an ‘expert mother’ (i.e. a mother of a child who had previously attended a referral appointment), using information booklets and SMS reminders, was effective in improving the uptake (Baum et al. 2019).

It is known that children with permanent HL may benefit from assistive hearing technology, for instance hearing aids, personal sound amplifiers or other ‘over-the-counter’ amplification products. Hearing aids are the best solution but need to be fitted properly to the user’s ears and hearing. The user must also be followed up with counselling and adjustment during the first period of use, and the hearing aid might need technical servicing, including change of batteries. All these components are known to be important for a successful introduction to wearing a hearing aid; thus, it is essential that hearing centres are readily available to achieve a good implementation. This is not the case in most LMICs; hence, hearing aids are not the most suitable technology.

Personal sound amplifiers do not need to be fitted individually, and therefore do not need the availability of local hearing centres to the same extent. Some studies also indicate that persons with mild to moderate HL might benefit from such equipment, even though an individually fitted hearing aid outperforms most personal sound amplifiers (Brody, Wu & Stangl 2018; Cho et al. 2019; Choi et al. 2020). It is known, however, that long reverberation times (RTs) and high background noise can compromise the sound quality from such devices (Wilson et al. 2011). Bad classroom acoustics have also been reported for several decades (Berg, Blair & Benson 1996; Fidêncio, Moret & Jacob 2014; Nábělek & Pickett 1974; Saravanan, Selvarajan & McPherson 2019; Wilson et al. 2020), and especially in LMICs, there is a lack of regulations and resources to improve the situation.

If treatment of common causes of HL (e.g. ear wax and infections) and simple interventions can be provided locally, either at the schools or in distributed centres, this could improve the situation for the children with preventable HL. The children with non-preventable HL will not benefit from such interventions and need other actions. To shed light on this, a study has been performed looking at three low-cost interventions to improve the learning situation for children where hearing aids are not a realistic alternative.

This project supports the United Nations (UN) Sustainable Development Goals: 1 (poverty), 3 (good health), 4 (quality education), 10 (reduce inequality) and 17 (partnerships for the goals). It also ensures user involvement and promotes the philosophies of ‘leave no one behind’ and ‘nothing about us without us’.

## Research methods and design

This study aimed to measure speech reception abilities in children with mild to moderate HL in their ordinary learning environments. As a result of the limited sample size, the study used a quasi-experimental design with within-group comparison of interventions. The study took place in the Kilimanjaro region in north-east Tanzania during two weeks in March 2020. Three schools (School A, B and C) were selected based on previous collaboration in the project. To gather sufficient information, each of these children were given a speech reception-in-noise test in a classroom with and without assistive hearing devices and in different positions in the classroom, according to the placement of a loudspeaker.

### Participants

A total of eight children participated, four girls and four boys, with mild to moderately severe HL from the three schools. These comprised all the children with permanent hearing impairment in the classes included in the study.

The children were selected through a basic hearing screening that consisted of otoscopy and air conducted pure tone audiometry. Children with impacted earwax or foreign bodies (e.g. insects, impacted sand and pebbles) in the ear canal and children with visible acute middle ear pathologies or pain were excluded from the study and referred to an ear specialist. All children had to be able to interpret and write numbers on a form to be included. Thresholds exceeding 25 hearing loss in decibels (dB HL) were considered a HL and both unilateral and bilateral losses were included. The children with hearing impairment had no previous experience with assistive hearing devices. The pure tone average (PTA4) for the eight children included in this study can be seen in Table 1. The audiograms for each child can be seen in Appendix 1.

**TABLE 1:** Pure tone average for the frequencies 500 Hz, 1 kHz, 2 kHz and 4 kHz.

School	Student	Age	Right ear (dB HL)	Left ear (dB HL)
School A	Student 1A	11	55	44
	Student 2A	11	8	74
	Student 3A	11	45	53
School B	Student 1B	13	38	31
	Student 2B	10	73	65
	Student 3B	14	20	40
School C	Student 1C	10	46	20
	Student 2C	10	56	66

dB HL, hearing loss in decibels.

### Control group

In addition to the children with hearing impairment, the teachers were asked to gather a control group of students at each school to fill the classrooms. The criteria for these students were to have no report of hearing problems and to be from the same academic year as the children with hearing impairment. In addition, they also had to be able to interpret and write numbers on a form to be included. These children were included in creating a situation closer to a normal class session, to normalise the acoustics and to be able to study how these students performed on the speech-reception test. These groups consisted of 40, 25 and 35 children at the three respective schools (A, B and C). All children were year 5 students, but their ages varied between 9 and 15 years.

### Testing environment

School A was a public school with approximately 400 boys and 360 girls. School B was a private Catholic school with approximately 150 boys and 130 girls. School C was a public school with approximately 260 boys and 290 girls and differed from the others by not having any electricity. The classroom construction was very similar in all schools, where the walls were made of cement blocks with a rendered paint finish and the floors were made of concrete. The roofing of all schools was angled, with corrugated iron sheets. Two of the schools (A and B) had flat ceilings made of fibreboard material, while one (School C) had corrugated iron roofing that had been left bare without any ceiling material.

#### Technical interventions

Two assistive listening devices were used in this study where both had a simple volume and tone control.

The first device was a Mino from Bellman & Symfon (called *personal amplifier* in this article), used with a pair of supra-aural headphones. It is possible to switch between omnidirectional and a directional microphone-mode with this device, but only the directional mode was used in this study because it is assumed to work best in reverberant conditions. The amplifier with the built-in microphone was placed on the child’s desk pointing at the speaker.

The second device was a Domino Classic from Bellman & Symfon (called *RM-system* in this article), which consists of a transmitter with a microphone that is worn by the teacher and a receiver with a pair of supra-aural headphones worn by the student. For the speech reception testing the microphone was hung around the loudspeaker and bags filled with fabrics were used to simulate a torso. This was carried out because the microphone is meant to be hung around the neck of the user.

The children were given the equipment the day before the speech-reception testing to try out and become familiar with the equipment. All children were given instructions on how to use it and could freely adjust the controls during the testing.

#### Speech recognition in noise-test

The children’s speech reception in the classroom was assessed using a beta-version of the digit triplet test (DTT) in Swahili. This test was developed during a bachelor thesis (Gjessing, Glesnes & Ørland 2020). The DTT is a closed-set audiometric speech test where digit triplets (e.g. 2-5-1) are presented in speech-shaped noise.

A loudspeaker that is designed to simulate a human talker was used to play the test signal (NTI TalkBox). The loudspeaker was placed in the middle of the front wall on a loudspeaker-stand about 1.5 m from the blackboard and 1.35 m above the ground pointing away from the blackboard. The speech-shaped noise was played back through a consumer radio (MusicBaby IPA-318) positioned on the floor pointing towards the blackboard. This was performed to let the noise signal be distributed as evenly as possible in the classroom.

The calibration of the loudspeakers was carried out with the sound level meter in one position, 1 m in front of the speech-signal loudspeaker. The DTT speech material, with silent intervals edited out, was used to calibrate the speech-signal loudspeaker. Calibration of the speech-noise loudspeaker was performed using the noise itself.

The speech signal was fixed at 65 dBA, which is a level between ‘normal’ (60 dBA) and ‘raised’ (66 dB) vocal effort, according to ISO 9921 (2003). This is in line with the results found by Sato and Bradley (2008) and Astolfi and Pallerey (2008), who investigated both female and male teachers’ vocal effort over a working day and found the average level to be 65.3 dBA. Bottalico and Astolfi (2012) found the level to be 62.1 dBA for female teachers. Two sound levels were used for the speech-shaped noise. Half of the DTTs used a noise level of 65 dBA and the second half used 70 dBA. This was carried out to avoid flooring and ceiling effects.

The children with hearing impairment performed one test list (22 digit triplets) while sitting in the front row centre in front of the loudspeaker without any personal hearing devices. Next, they all moved to the outermost seats in the classroom, either to the front row right or in the back row centre or left. In this position they performed one test list with the personal amplifier, the RM-system and without any amplification.

The children with normal hearing also participated in the testing and were used as a control group – one group in each classroom. These children were sitting in the same position for the whole test except for the children who swapped seats with the participants with hearing impairment. All participants responded nonverbally by writing down all the digits in the digit triplet that they could perceive on an answer sheet.

#### Measurement of the room acoustics

Acoustical variables measured in the classrooms included RT, background noise and speech transmission index (STI). The RT was measured following the guidelines of the engineering method described in ISO 3382-2 (2008). Six different combinations of microphone and speaker placements were recorded and used to calculate mean RT for all frequencies. To get a single value for each classroom, a mean was calculated using the six 1/3 octave bands between 400 Hz and 1250 Hz.

The background noise was measured using a Norsonic NOR-140 sound level meter placed in the middle of the classrooms. The measurements were performed in empty classrooms during a normal school day while normal classes were being held in the rest of the school.

The speech transmission index was measured following the recommendations in the standard IEC 60268-16 (2020) for measurements using the *Speech Transmission Index for Public Address Systems* (STIPA) method. The loudspeaker was placed in the same position as in the speech recognition in noise-test. Four positions in each classroom were measured: front row right and centre and back row left and centre, viewed from the teacher’s perspective. A mean was calculated using the results from these four measurements. The classroom was empty during the measurements.

With the physical measurements of the rooms and the results from the RT measurements, each classroom’s critical distance was calculated. The critical distance is the point in a room where the level of the direct sound from the sound source and the level of the reflected, reverberant sound is equal (Crandell & Smaldino 2000).

### Data analysis

The statistical analysis was performed using the Statistics and Machine Learning Toolbox in Matlab (MathWorks 2021). A paired *t*-test was used to compare the results from the DTT for the different interventions. A Bonferroni correction was applied to the *p*-value limit.

### Ethical considerations

This study was approved by Norwegian Centre for Research Data (reference number 58283) and the National Institute for Medical Research (NIMR) in Tanzania (reference number NIMR/HQ/R.8a/Vol.IX/3009). The head of school at each school was introduced to the project and signed a consent form on behalf of the participating children’s guardians. The mandate to do this was given by the district’s education officer. It was voluntary to participate and the children were free to withdraw from the project at any given time.

## Results

A description of the classrooms in the study can be found in Table 2, including dimensions and the acoustical parameters.

**TABLE 2:** Classroom description.

School	Width (m)	Length (m)	Height (m)	Reverberation time (s)	Critical distance (m)	Background noise (dBA)	STI	s.d	*n*
School A	6.3	9.0	3.2	1.4	0.91	52.2	0.42	0.05	4
School B	7.7	9.8	2.8	1.4	0.98	43.3	0.48	0.04	4
School C	6.0	9.1	3.1, *4.0	0.8	1.23	47.8	0.56	0.06	4

* height under ridge.

STI, speech transmission index; s.d, standard deviation.

Table 3 shows the mean DTT scores for the students with normal hearing (two sequential desk-rows with two students at each desk) in different positions in the classrooms. As expected, the mean SRT score for the front row centre position had the highest mean with the smallest spread of scores in all three schools. A less obvious finding was that front row right position had a lower mean than the back row centre position in schools B and C. The distance between the loudspeaker and the student’s desk in the back row centre position was longer than between the loudspeaker and the front row right position in all three schools, which highlights that the angle between the talker and the listener influences the speech perception. In both schools B and C, the back row left position had the lowest mean score.

**TABLE 3:** Digit triplet test scores for students with normal hearing in different positions in the classrooms at the different schools. The results are the mean value of four children in each position, with standard deviations in brackets.

Position	School A	School B	School C
Front row centre	98.5% (1.3)	94.5% (3.1)	99.8% (0.5)
Front row right	85.0% (5.5)	60.8% (3.9)	70.3% (14.1)
Back row centre	78.5% (15.9)	68.3% (12.0)	92.5% (7.7)
Back row left	81.0% (5.9)	50.8% (13.0)	67.3% (5.5)

Because of differences in the room acoustics between the classrooms used in the experiments, the DTT results collected at the different schools were analysed separately.

**FIGURE 1:**
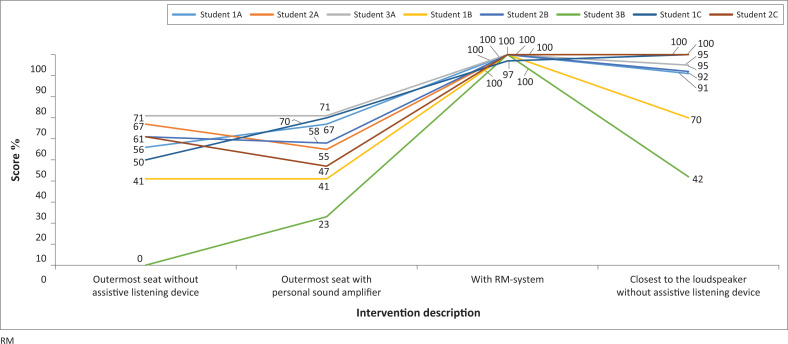
Digit triplets test scores for the eight students with hearing impairment at the three schools.

Figure 1 shows the DTT score for each of the eight students with hearing impairment. With the RM-system, all test subjects scored 100%, except one who scored 97% on the test, regardless of the position in the classroom. Because of this saturation, the RM-system was removed from the statistical comparison of groups, but this intervention outperforms all the others with close to full score for all the children with hearing impairment.

A paired *t*-test showed that when the students were seated in the outermost seats in the classroom, the use of personal amplifier (*M* = 54.0, *s.d.*= 16.52) did not improve the results from the situation without an assistive listening device (*M* = 50.9, *s.d.*= 22.62); *t*(7) = –0.6434, *p* = 0.54. When the student moved closer to the speaker (*M* = 85.6 *s.d.*= 20.02), there was a significant improvement compared with no personal amplifier: *t*(7) = –11.50, *p* < 0.001 and compared with personal amplifier: *t*(7) = –8.28, *p* < 0.001.

### Limitations

Even though this study aimed at preserving an ecologically valid situation, where the children performed a speech reception test in a familiar context of the classroom surrounded by their classmates, there are several limitations.

Firstly, the number of participants with hearing impairment was small (only eight children). This makes it difficult to draw any strong conclusions, and the results should be viewed as indications. Nonetheless, the statistical analysis did show significant improvements of the speech recognition for two of the interventions.

Next, all the children with hearing impairment had the possibility to adjust the volume and tone control of the devices during the test. The settings were not inspected, so it is possible that some of the children had misadjusted their devices. This is, however, a realistic scenario for these devices. Both devices were also found to have at least 5 dB – 10 dB amplification (not shown here), even on the lowest volume setting, so all children had at least some amplification during the testing.

Furthermore, inclusion of the children with hearing impairment’s classmates who participated was based on self-report and no audiologic testing. This means that the children in the control group could also have some degree of HL without knowing it. If so, the DTT scores for the control group could be somewhat higher.

Finally, the acoustical differences in the testing environments make it difficult to compare data collected in the different classrooms. However, the acoustical properties of the classrooms were measured and are reported.

## Discussion

In this study, speech recognition using a DTT in Swahili was used to measure the effect of different interventions that can be implemented in schools to improve the learning situation for children with hearing impairment. The three interventions studied are presented here.

Firstly, the simplest measure, where the student is moved closer to the teacher, can improve speech perception and therefore can lead to a better learning environment. All the children improved their results with this intervention, and six out of eight got a score above 90%. This will, however, only work if the teacher is aware of the challenge and tries to be close to the student(s) with hearing impairment during teaching. A challenge is that teachers often have to move around in the classroom and therefore cannot maintain a close distance all the time. Another challenge is that it can be difficult for the students with hearing impairment to hear the other students who are not sitting close to them; this can lead to exclusion from dialogues.

Secondly, the use of a simple personal amplifier has clear limitations in classroom settings with bad room acoustics. As a result of the long RTs, the amplifier will only work when the user is within the critical distance to the speaker. This distance was calculated to be from approximately 0.91 m – 1.23 m, which is very short. As we found that moving closer to the speaker will improve the situation by itself, it is not obvious that a personal amplifier will give any additional benefit. This is something that should be studied further.

Thirdly, the RM-system gave the best speech recognition among the interventions that was tested. That an RM-system outperforms personal amplifiers, hearing aids and cochlear implants in gaining increased speech perception in classroom situations has been demonstrated previously (Zanin & Rance 2016); this indicates that RM-systems also can provide benefit to children with mild to moderate HL. Because of the wireless transmission of the speech, the student will hear the teacher regardless of where they are seated in the classroom. The teacher must, however, use the microphone for this system to work, and both the teacher and the user must also have the competence to use the device. Rekkedal (2014) has looked at factors affecting the use of technical interventions and found that the teachers’ attitude towards microphones was most important. She also found that the teachers in her study felt they needed more knowledge about hearing impairment. This means that training is essential and knowledge of the significant benefit this can give must be clearly stated to promote usage.

There are also some challenges associated with RM-systems. As the signal is provided to the user using a microphone, other students in the class also must have microphones to be heard. This can be solved by having one or more handheld microphones that can be passed around the classroom to the talker, but this further complicates both the use and the technical competence needed. Even if some of the RM-systems also have microphones in the device that can be switched on if needed (for instance when other students are talking), the long RT in the classroom will also affect these. This is the same challenge as with the personal amplifier mentioned here.

Common for all the interventions is that education must be given to ensure that they are implemented in the best way. This information must contain both general information about the challenges associated with HL and also guidance on how the people around (i.e. teachers, other students, guardians) can accommodate it. For the technical devices, training of both the user and the technical staff providing service of the devices is also necessary.

It must also be observed that only mild to moderate hearing impairments were looked at in this study and that the HL was quite different among the children. The speech recognition results also had little correlation with the severity of the HL. A reason could be that some of the children had other disabilities, such as cognitive impairment, but this was neither screened for nor investigated any further. Two of the eight participants did not achieve the same benefit as the others. These two children were those who scored the lowest on all tests, indicating that they had greater challenges with speech perception than the others. Even if this could be related to other impairments, these children came from School B, where the control group also scored lower than the other schools. This could indicate that the room was more challenging than the other rooms.

The study did not look at personal amplifier use in the position close to the speaker, and therefore it is not possible to say if this could further improve the listening situation for the children. It does, however, show that if personal amplifiers are introduced in a school setting, the teachers must be given knowledge on how to best utilise these devices. If the children were provided with such a device and seated in the back or at the side of the classroom, these results indicate that there is a chance that the students will hear better without the equipment.

Mealings (2016) reviewed national and international standards and recommendations of classroom acoustic conditions and found recommended noise levels ranging from 25 dBA to 50 dBA, recommended RTs ranging from 0.3 s to 0.9 s and STI values ranging 0.6–0.75 for developing children. For children with hearing impairments and language delays, the recommended values were noise levels lower than 20 dBA – 35 dBA and RTs shorter than 0.3 s – 0.7 s.

The room acoustical measurements in this study showed that none of the classrooms met recommendations from international standard. Two of the classrooms had RTs above 1.3 s and clearly show a major challenge for learning in these schools. This affects all students, not only those with hearing impairment, but those who also have hearing challenges will suffer more. An observation made from the control group results is that School B did worse than School A on the DTT, even though the acoustical conditions were measured to be better in School B. If this observation is true, it might be a challenge for acoustical treatments of classrooms and something that should be studied further.

To further elucidate the potential in these low-cost interventions, more research is needed on the effect. Student performance after different interventions should be studied and cost–benefit analysis should be performed to appraise them.

## Conclusion

This study, although small in sample size, showed that personal amplification with directive microphones gave little to no effect in assisting the children with hearing impairment. One of the main reasons is the challenging acoustical conditions in the classrooms that compromise the sound quality in such equipment. The best speech perception was achieved using an RM-system that circumvents the bad acoustical conditions by using microphones close to the speaker and transmitting the sound wirelessly to the user. Interestingly, the results also indicate that the children with hearing impairment could get good benefit simply by moving closer to the teacher. This is a low-cost alternative but will require proper training of both the child with hearing impairment, the teacher, the other students and the guardians in order to work. The effect of such intervention must, however, be studied further.
